# Comparison of Depression, Stress, Anxiety, Cognitive Status, and Quality of Life Before and After Renal Transplantation

**DOI:** 10.7759/cureus.71983

**Published:** 2024-10-21

**Authors:** Sudip S Mukherjee, Suprakash Chaudhury

**Affiliations:** 1 Psychiatry, Dr. D. Y. Patil Medical College, Hospital and Research Centre, Dr. D. Y. Patil Vidyapeeth, Pune, IND

**Keywords:** anxiety, chronic kidney disease, cognitive impairment, depression, end-stage renal disease (esrd), quality of life, renal transplant

## Abstract

Introduction: Chronic kidney disease is a prevalent and life-altering condition that significantly impacts quality of life. Kidney transplantation (KT) is an effective treatment for CKD, but it may also trigger psychological distress, including depression, anxiety, and cognitive impairment. This study aims to compare the levels of depression, anxiety, stress, cognitive status, and quality of life in KT recipients before and after surgery.

Methods: This descriptive, qualitative comparative study included 100 patients scheduled for renal transplantation at a tertiary care center in Pune, India. Participants were evaluated before and one month after the surgery using the Depression Anxiety Stress Scale-21 (DASS-21), Montreal Cognitive Assessment (MoCA), and Kidney Disease Quality of Life Short Form, Version 1.3 (KDQOL SFv1.3) scales. The Wilcoxon signed-rank test and multiple regression analysis were applied to identify predictors of psychological outcomes.

Results: The study found a significant reduction in depression and cognitive impairment posttransplant. Depression decreased from 19% pretransplant to 13% posttransplant. Anxiety and stress also showed slight reductions, while cognitive impairment decreased from 29% to 19%. Additionally, there was a notable improvement in 15 out of the 17 dimensions of quality of life, particularly in physical functioning and social interactions.

Conclusion: KT significantly improves depression, cognitive function, and overall quality of life in CKD patients, though psychological challenges may persist. Ongoing mental health support is crucial for these patients.

## Introduction

Chronic kidney disease (CKD) is characterized by diminished renal function, typically indicated by levels of glomerular filtration rate, serum creatinine concentration, and proteinuria, and often assessed using the albumin-to-creatinine ratio [1,2]. In 2016, CKD impacted 753 million individuals globally, including 417 million women and 336 million men [3]. Cardiovascular disease, end-stage renal disease (ESRD), and mortality are adverse outcomes linked with CKD. Given the numerous challenges that patients must contend with, receiving a diagnosis of a chronic condition such as ESRD can lead to a significant amount of stress for the patient. ESRD is a life-altering condition that can limit or put an end to one's professional life, hindering the performance of existing social roles. Alterations in usual behaviors; diminished tasks associated with daily living; hypersensitivity, especially to strangers; bewilderment; and a feeling of helplessness are some of the behavioral and emotional changes commonly seen in these patients [4]. As a result, anxiety, depression, and cognitive impairment are some of the most common mental health conditions in patients with ESRD.

Given the short life expectancy and heavy symptom burden experienced by many older persons with renal disease, health-related quality of life (HRQOL) is identified as a crucial patient-centered outcome [5]. Patients with CKD often experience a decline in their HRQOL [6]. Patients with ESRD have a decreased HRQOL, which is now widely recognized [7-11]. Various parameters like sociodemographic (age, sex, literacy, and income), clinical (presence of comorbidity, albumin levels, and hemoglobin levels), and psychological (depression, anxiety, and personality) factors have been frequently linked to HRQOL in such patients [12-14].

Kidney transplantation (KT) is the preferred treatment for renal failure and CKD because of its effectiveness and positive health outcomes across the world, and India is no exception [15]. Enhancing bodily functions and boosting QOL are two areas where KT has a strong track record of success [16]. Prolonged graft function and enhanced HRQOL are probable outcomes of effective therapy [17]. Despite the advantages of receiving a transplant, it necessitates psychological adaptation and presents unique treatment obstacles that may evoke negative emotions like anxiety, remorse, and overall distress and, hence, may lead to depression [18]. Depression plays a key role in several poor clinical outcomes in KT recipients. It has been seen in previous studies that depression may increase the risk of nonadherence to an immunosuppressive regimen [19].

Research studying depression, anxiety, stress, cognitive status, and QOL in KT patients has been carried out previously, but only a few focused on the changes that take place throughout the transplant process, and even fewer studies have been carried out in India. The primary goal of this study was to examine and contrast the levels of depression, anxiety, stress, cognitive function, and QOL among KT recipients both before and after their surgery.

## Materials and methods

This descriptive, qualitative, comparative study was done at a tertiary care center affiliated with Dr. D. Y. Patil Medical College, Hospital and Research Centre, located in an urban area in Pune, Maharashtra. The study proposal was submitted to the institutional ethics committee, and the study commenced upon receiving approval (approval no. IESC/PGS/2022/54, dated 28/09/2022). This was carried out from August 1, 2022, to July 31, 2024.

Adult patients undergoing renal transplant at a tertiary care center in Pimpri, Pune, were invited for the study. Written informed consent was obtained from the patients who participated in the study, and sociodemographic variables were collected using a self-reported questionnaire.

Sample size calculation

According to an earlier study, the prevalence of depression in renal transplant recipients was 7% [20]. The Fisher’s formula was used: $n = [z^{2}*p*(1-p)]/d^{2}$, where n is the required sample size, z is the confidence level at 95% (1.96), p is the estimated prevalence (7%), and d is the margin of error at 5% (0.05). Substituting these values, we have n = 1.96x1.96x 0.07(0.93)/0.05x 0.05. This calculation resulted in a sample size of approximately 100.

Inclusion and exclusion criteria

The patients included in our study were those who were scheduled for renal transplant, had given consent to participate in the study, and were clinically stable to undergo the interview. Patients with a history of psychiatric illness, who were on dialysis, and who were below 18 years of age were not included. 

Data collection tools

Sociodemographic proforma, including name, age, sex, education, occupation, and history of substance use, among others, and lab parameters of the participants were recorded before the transplant process. The Depression Anxiety Stress Scale-21 (DASS-21) was used to measure the levels of depression, anxiety, and stress in patients undergoing KT, through self-reported scales [21]. Studies confirm its high reliability, with Cronbach's α scores between 0.74 and 0.93.

Given the subtle cognitive challenges experienced by KT patients, the Montreal Cognitive Assessment (MoCA) scale was used to capture early signs of impairment that other tools, like the Mini-Mental State Examination (MMSE), might miss [22]. To identify mild cognitive deficits in patients, we employed the MoCA, a tool proven to have a robust test-retest reliability (intraclass correlation coefficient = 0.87).

The Kidney Disease Quality of Life Short Form, Version 1.3 (KDQOL SFv1.3) is a self-report measure developed for individuals with kidney disease and on dialysis [23]. It includes 43 kidney disease-targeted items as well as a 36-Item Short Form Health Survey (SF-36) as the generic core. Dialysis staff encouragement and patient satisfaction subscales were not applied, as none of our subjects was on dialysis. Kidney disease scales exhibited desirable internal consistency (Cronbach's α 0.822-0.906) and item-to-scale correlation (range 0.763-0.903), and a three-factor model fit the data well (comparative fit index 0.934, root mean square error of approximation 0.085).

One hundred patients scheduled for renal transplantation were recruited. They were interviewed using a semi-structured questionnaire to gather sociodemographic variables. Written informed consent was obtained from all patients enrolled in the study. Levels of depression, anxiety, and stress were evaluated using the DASS-21, cognitive function was measured using the MoCA, and QOL was assessed through the KDQOL SFv1.3, both one month before and one month after KT.

The data was analyzed using the IBM SPSS Statistics for Windows, Version 26.0 (Released 2019; IBM Corp., Armonk, New York, USA). The Wilcoxon signed-rank test was used to determine the significance after transplants. A chi-square test was done. Multiple regression analysis was done to determine the predictors. Spearman’s correlation analysis was done to detect correlations.

## Results

A total of 100 subjects who were scheduled for renal transplantation surgery were recruited after obtaining informed consent and were interviewed before and one month after the surgery. Among them, 65 were males while 35 were females. The mean age was 36.07 (SD ± 10.14). Of the study participants, 84 were from a nuclear family, while 16 were from a joint family. Most of our participants belonged either to the lower middle (50) or upper middle (43) socioeconomic status. Only one participant belonged to the upper class, and six belonged to the upper lower class. None of the participants was living alone. Twelve participants were unmarried, while 88 were married. History of substance use was found in only 18 participants. Ninety participants have hypertension, which was the most common comorbidity, with seven of them also having type 2 diabetes mellitus. Type 2 diabetes alone was seen in three of the participants. Seventy-one participants received a living-donor kidney, while 29 received a deceased-donor kidney. Sociodemographic characteristics were collected from all patients and are shown in Table 1. 

**Table 1:** Sociodemographic variables of patients undergoing KT KT: kidney transplantation.

Sociodemographic variables	Frequency (N)
Age	
18-35 years	59
36-55 years	37
>56 years	4
Religion	
Hindu	79
Muslim	11
Christian	10
Type of residence	
Urban	92
Rural	8
Type of family	
Nuclear	84
Joint	16
Socioeconomic status	
Upper	1
Upper middle	43
Lower middle	50
Upper lower	6
Marital status	
Married	88
Unmarried	12
Substance use	
Present	18
Absent	82

Table 2 provides the mean (±SD) of the results of the lab parameters of renal transplant recipients before the surgery. The mean hemoglobin and calcium levels were low. Meanwhile, the mean urea, creatinine, urine protein creatinine ratio (UPCR), and parathyroid hormone (PTH) levels of the participants were elevated.

**Table 2:** Mean (±SD) of lab investigations of renal transplant recipients

Lab parameters	Mean	SD
Hemoglobin	8.245	1.14441
White blood cell	6493	2612.93
Platelet count	164880	48684.35
Urine protein creatinine ratio	3.2956	1.079432
Urea	93.17	44.32856
Creatinine	6.1996	4.11836
Uric acid	6.3685	2.289361
Sodium	137.02	3.069137
Potassium	4.0692	0.672072
Bicarbonate	26.399	2.16192
Calcium	8.151	0.564711
Phosphate	5.5796	1.502808
Parathyroid hormone	509.099	140.6774
Total protein	6.72	0.564801
Albumin	3.478	0.487151

Depression, anxiety, stress, and cognitive functions before and after KT

The mean (±SD) DASS-21 and MoCA scores of the study subjects are given in Table 3. The mean scores of all self-report scales of DASS-21 and MoCA improved after the transplant, indicating the beneficial effects of the transplant process on CKD patients. Significant improvement was observed for the DASS-d (5.18 (±4.31) to 4.44 (±3.83)) and MoCA (25.76 (±2.49) to 26.62 (±1.87)) scores upon renal transplantation.

**Table 3:** Comparison of the DASS-21 and MoCA scores before and after renal transplantation *Significant at p < 0.05. DASS-d: Depression Anxiety Stress Scale-depression, DASS-a: Depression Anxiety Stress Scale-anxiety, DASS-s: Depression Anxiety Stress Scale-stress, MoCA: Montreal Cognitive Assessment Scale.

	Pretransplant		Posttransplant		Wilcoxon signed-rank test	Asymp. Sig.*
	Mean	SD	Mean	SD		
DASS-d	5.18	4.31	4.44	3.83	-4.007	0.000
DASS-a	3.76	2.72	3.42	2.88	-1.588	0.112
DASS-s	8.82	5.38	8.46	4.8	-1.842	0.66
MoCA	25.76	2.49	26.62	1.87	-6.272	0.000

Table 4 shows that 13 and six subjects during the pretransplant phase had mild and moderate depressive scores on the DASS-21, respectively, compared to eight and five in the posttransplant phase. Nine and five subjects during the pretransplant phase had mild and moderate anxiety scores on the DASS-21, respectively, compared to six and five in the posttransplant phase. Nine and five subjects during the pretransplant phase had mild and moderate stress scores on the DASS-21, respectively, compared to six and five in the posttransplant phase. Twenty-nine subjects during the pretransplant phase showed mild cognitive impairment on the MoCA, which reduced to 19 in the posttransplant phase.

**Table 4:** Comparison of the distribution of participants according to the DASS-21 and MoCA scores before and after the transplant *Significant at p < 0.05. DASS-d: Depression Anxiety Stress Scale-depression, DASS-a: Depression Anxiety Stress Scale-anxiety, DASS-s: Depression Anxiety Stress Scale-stress, MoCA: Montreal Cognitive Assessment Scale.

	Pretransplant	Posttransplant	Chi-square test	p-value*
DASS-d				
Normal	81 (81%)	87 (87%)	1.4957	0.473
Mild	13(13%)	8 (8%)		
Moderate	6 (6%)	5 (5%)		
DASS-a				
Normal	86 (86%)	89 (89%)	0.6514	0.722
Mild	9 (9%)	6 (6%)		
Moderate	5 (5%)	5 (5%)		
DASS-s				
Normal	77 (77%)	80 (80%)	1.8573	0.395
Mild	19(19%)	19 (19%)		
Moderate	4 (4%)	1 (1%)		
MoCA				
Normal	71 (71%)	81 (81%)	2.7412	0.978
Mild cognitive impairment	29 (29%)	19 (19%)		

QOL before and after KT

All domains of the KDQOL SFv1.3 showed improvement after the renal transplant (Table 5). Compared to the pretransplant phase, statistically significant differences were observed in the posttransplant phase in terms of symptom list, effects of kidney disease, burden of kidney disease, cognitive status, quality of social interaction, sleep, and social support dimensions of kidney disease-targeted areas. Similarly, statistically significant improvement was also observed in all domains of the SF-36 subscales of the KDQOL SFv1.3, one month after the renal transplant.

**Table 5:** Comparison of the kidney disease-targeted areas and SF-36 of the KDQOL SFv1.3 before and after the renal transplant *Significant at p < 0.05. SF-36: 36-Item Short Form Health Survey, KDQOL SFv1.3: Kidney Disease Quality of Life Short Form, Version 1.3.

Kidney disease-targeted areas	Pretransplant		Posttransplant		Wilcoxon signed-rank test	Asymp. Sig.*
	Mean	SD	Mean	SD		
Symptom list	68.41	7.56	73.57	7.40	-8.106	0.000
Effects of kidney disease	68.66	8.95	76.09	7.48	-8.051	0.000
Burden of kidney disease	47.94	15.21	50.13	13.40	-4.831	0.000
Work status	13.5	29.39	18.0	33.56	-2.251	0.24
Cognitive status	77.6	10.95	83.2	11.21	-8.074	0.000
Quality of social interaction	85.33	8.10	90.4	7.793	-5.620	0.000
Sexual function	21.75	39.31	23.5	40.09	-2.220	0.26
Sleep	79.28	12.45	80.25	11.81	-3.130	0.002
Social support	88.98	10.93	94.32	7.91	-5.657	0.000
SF-36						
Physical functioning	55.1	14.77	61.7	10.74	-7.353	0.000
Role-physical	20.75	24.11	31.25	35.06	-5.651	0.000
Pain	64.5	12.58	68.1	14.91	-4.902	0.000
General health perception	53.95	14.11	56.75	15.43	-6.048	0.000
Emotional well-being	73.52	6.93	76.96	7.98	-6.565	0.000
Role-emotional	50.0	20.3	57.33	21.23	-2.515	0.000
Social function	55.13	14.0	58.75	16.99	-5.038	0.000
Energy/ fatigue	57.1	14.98	59.4	17.38	-4.645	0.000
Overall health rating	60.39	8.62	64.76	9.48	-8.641	0.000

Multiple regression analysis revealed the factors influencing the DASS-21 scores and QOL scores of subjects before undergoing a renal transplant (Table 6). Sodium levels were positive predictors, while pretransplant effects of kidney disease and pain items of the KDQOL SFv1.3 were negative predictors of pretransplant depression scores of the DASS-21. Pretransplant stress scores of the DASS-21 and receiving a deceased-donor kidney were positive predictors of pretransplant anxiety scores, while sexual function items of the KDQOL SFv1.3 and serum creatinine were negative predictors. Furthermore, pretransplant anxiety scores of the DASS-21, UPCR levels, sexual function items of the KDQOL SFv1.3, and the presence of comorbidities were revealed to be positive predictors of pretransplant stress scores. Symptom list item of the KDQOL SFv1.3 before transplantation was a negative predictor of pretransplant stress scores of the DASS-21.

**Table 6:** Multiple regression analysis for the predictors of pretransplant DASS-21 *Significant at  p < 0.05. DASS-d: Depression Anxiety Stress Scale-depression, DASS-a: Depression Anxiety Stress Scale-anxiety, DASS-s: Depression Anxiety Stress Scale-stress, VIF: variance inflation factor.

Predictors	Independent variables	Unstandardized coefficients		Standardized coefficients	t	Sig	95.0% CI for B		Collinearity statistics	
		B	Std. error	Beta			Lower bound	Upper bound	Tolerance	VIF
DASS-d	Effects of kidney disease	-.171	.047	-.355	-3.596	.001	-.265	-.077	.700	1.429
	Pain	-.091	.034	-.267	-2.699	.008	-.159	-.024	.697	1.434
	Sodium	.233	.116	.167	2.007	.048	.003	.463	.989	1.011
DASS-a	DASS-s	.216	.047	.428	4.627	.000	.123	.309	.920	1.087
	Sexual function	-.020	.006	-.293	-3.176	.002	-.033	-.008	.923	1.083
	Creatinine	-.146	.060	-.222	-2.434	.017	-.265	-.027	.942	1.062
	Type of donor	1.159	.531	.195	2.184	.031	.105	2.213	.989	1.012
DASS-s	Symptom list	-.317	.057	-.446	-5.588	.000	-.430	-.204	.920	1.086
	DASS-a	.614	.156	.310	3.936	.000	.304	.923	.945	1.058
	UPCR	.818	.399	.165	2.052	.043	.027	1.610	.906	1.104
	Sexual function	.027	.011	.194	2.415	.018	.005	.048	.907	1.103
	Comorbidity	1.510	.713	.172	2.117	.037	.094	2.926	.891	1.122

The correlation analyses revealed pretransplant depression (rs = 0.748), anxiety (rs = 0.294), stress (rs = 0.432), and social support items (rs = 0.270) to be positively associated with posttransplant depression. Meanwhile, posttransplant depression correlated negatively with symptom List (rs = -0.492), effects of kidney disease (rs = -0.587), burden of kidney disease (rs = -0.368), cognitive status (rs = -0.304), physical function (rs = -0.426), role-physical (Rs = -0.377), pain (rs = -0.346), general health perception (rs = -0.341), emotional well-being (rs = -0.327), role-emotional (rs = -0.312), social function (rs = -0.354), and energy/fatigue (rs = -0.393) items before transplantation.

The correlation analyses demonstrated that posttransplant anxiety correlated positively with pretransplant anxiety (rs = 0.641) and stress (rs = 0.275). Symptom list (rs = -0.311), effects of kidney disease (rs = -0.336), burden of kidney disease (rs = -0.327), physical function (Rs = -0.346), role-physical (rs = -0.289), pain (rs = -0.230), general health perception (rs = -0.373), and social function (rs = -0.425) items of the KDQOL SFv1.3 before transplantation correlated negatively with posttransplant anxiety.

Furthermore, posttransplant stress correlated positively with pretransplant depression (rs = 0.274), anxiety (rs = 0.293), and stress (rs = 0.911). Among the KDQOL SFv1.3 domains before transplantation, posttransplant stress correlated negatively with symptom list (rs = -0.521), effects of kidney disease (rs = ­0.422), sleep (rs = -0.315), physical function (rs = -0.321), role-physical (rs = -0.280), pain (rs = -0.388), general health perception (rs = -0.385), and social function items (rs = -0.472). They were all significant at p < 0.01.

## Discussion

In our study, among the 100 study participants who were scheduled for renal transplant, 65 were males, while 35 were females. This finding is in line with the findings of previous Indian studies [15,24,25]. Hypertension was the most prevalent comorbidity (90 out of 100) in our study population. Seven out of the 90 participants who have hypertension also have type 2 diabetes mellitus. Similar findings have also been reported in an Indian study [26] and a South Korean study [27].

In our study, 19% of the transplant recipients showed signs of depression before KT. In CKD, depression can be present in 15%-60%, depending upon the use of various self-report questionnaires, which is congruent with our findings [28]. After KT, the prevalence of depression dropped to 13%. This is in line with the findings of other research. Using the Beck Depression Inventory, another descriptive, analytical study conducted in 2014 assessed and contrasted the prevalence of depression before transplants, upon discharge following the transplant, and three months following the transplant. Mild to very severe depression was prevalent in 70.6%, 56.9%, and 52.9% of the patients before, during, and three months after KT, respectively [29]. Recent research also reported similar findings. A Dhaka-based study reported a prevalence of depressive disorder in 51% of patients before KT, which dropped to 28.9% after KT [30].

Of the participants, 14% and 23% showed symptoms of anxiety and stress, respectively, before KT, which reduced to 11% and 20% after KT. The association between CKD/ESRD and anxiety has been less studied when compared to depression. Literature shows varied prevalence of anxiety in patients with ESRD, ranging from 12% to 52% [31-32]. Our findings were contradictory to the findings of Müller et al. who observed an increase in the levels of anxiety from 21.6% to 25.0% after transplantation using the Hospital Anxiety and Depression Scale-Anxiety [33].

A multiple regression analysis was performed to identify factors influencing the DASS-21 scores in subjects before renal transplantation. Sodium levels positively predicted pretransplant depressive scores. There is evidence suggesting that depression is linked to a significant rise in residual sodium levels, either due to reduced excretion or increased retention of water and sodium without any net gain or loss during the illness [34]. This could explain how sodium could have played a role in predicting depressive scores in our study. Various items of the KDQOL SFv1.3 were predictors of the DASS-21. The effects of kidney disease and pain items negatively predicted depressive scores. Pretransplant stress scores on the DASS-21 and receiving a deceased-donor kidney positively predicted pretransplant anxiety scores. Sexual function items and serum creatinine levels negatively predicted anxiety scores. Pretransplant anxiety scores on the DASS-21, UPCR levels, sexual function items, and the presence of comorbidities positively predicted pretransplant stress scores. Symptom list items from the KDQOL SFv1.3 negatively predicted pretransplant stress scores on the DASS-21.

Twenty-nine percent of the subjects had mild cognitive impairment before KT, which reduced to 19% after KT. Murray et al. [35] and Kalirao et al. [36] observed a higher prevalence of 87% and 75% of cognitive impairment in hemodialysis and peritoneal dialysis patients, respectively. KT has proven to improve cognitive status in ESRD patients, as seen in our study. Gupta et al. reported improvement in Logical Memory I and II, Digit Symbol Substitution Test scores, and Category Fluency Test in 78 individuals after KT [26]. One study also reported improvement in the MMSE [37]. One possible explanation for the improvement after KT is that it facilitates the removal of inflammatory mediators and intermediate molecules, including tumor necrosis factor, interleukin (IL)-6, and IL-1β, which are challenging to eliminate through dialysis [38].

In our study, posttransplant QOL improved in all recipients after KT compared to their pretransplant status. Significant improvement in 15 of the 17 dimensions of the KDQOL SFv1.3 was observed. Kostro et al. also reported significant improvements in 14 of the 19 dimensions of the KDQOL SFv1.3 after KT [39]. Czyżewski et al. also reported significant differences in physical functioning, effects of kidney disease, burden of kidney disease, work status, sleep, and overall health rating when comparing the HRQOL of patients on hemodialysis and peritoneal dialysis with that of individuals three months after the transplant [40].

Our study found that there were no correlations of depression, anxiety, or stress with any of the sociodemographic characteristics. Mosleh et al. [41] and Müller et al. [33] reported no significant correlations of anxiety with any of the sociodemographic variables. However, Mosleh et al. observed depression to be correlated only with age (p = 0.003) [41].

Limitations

This study did have some limitations. This study was conducted at a single center, but a multicenter study was preferred. The number of subjects enrolled in our study was relatively small. Hence, the findings of the study may require validation on a larger scale. Since this was a hospital-based study, there are limitations to generalizing these findings.

## Conclusions

In our study, we observed a significant improvement in depression, cognitive impairment, and various aspects of the QOL one month after renal transplantation, highlighting its benefits in patients with CKD. Although KT provides much relief to the patient, psychiatric comorbidities remain; hence, clinicians should have a high index of suspicion while treating such patients. Psychiatric evaluation, pretransplant counseling, and psychoeducation should be essential. However, further research should not only focus on monitoring long-term outcomes but also explore specific intervention methods that address both psychological and cognitive challenges in posttransplant patients. Future studies should also investigate the effectiveness of targeted therapies, such as cognitive-behavioral therapy, mindfulness-based interventions, and pharmacological approaches, in managing depression, anxiety, and cognitive decline after transplantation. Understanding the optimal timing and combination of these interventions would help clinicians support better mental health outcomes in these patients. Moreover, it is crucial to develop comprehensive clinical guidelines that incorporate mental health support both before and after transplantation. Recommendations should include routine psychiatric assessments, ongoing psychological support, and tailored interventions to address the unique challenges faced by transplant recipients. This can help us maximize the benefits of KT on patients' overall well-being, ensuring improved QOL in both the short and long terms.
